# Organizational cultures in the Swedish restaurant business and the risk for sexual harassment

**DOI:** 10.1093/eurpub/ckac131.269

**Published:** 2022-10-25

**Authors:** K Gillander Gådin, K Zampoukos

**Affiliations:** 1 Mid Sweden University, Department of Health Sciences, Sundsvall, Sweden; 2 Mid Sweden University, Department of Economics, Geography, Law and Tourism, Östersund, Sweden

## Abstract

**Background:**

The hospitality sector has the highest level of sexual harassment incidents compared to any other sector. The negative consequences of sexual harassment at the restaurant workplace are not limited to the health of the victim alone as it also affects the organization as well as the health of a society. The organizational context is a fundamental determinant of sexual harassment and we need to increase our understanding of organizational cultures that affect such behaviors in order to develop and implement effective interventions in the restaurant business. The aim of this study was to give a comprehensive picture of organizational cultures that increase the risk for sexual harassment in the restaurant business.

**Methods:**

Individual interviews with twenty-nine employees in the restaurant business (e.g. waiting staff, chefs, bartenders, managers) were conducted during 2019-2021. Thematic analysis was used to analyze organizational cultures that increase the risk for sexual harassment in the restaurant business. Preliminary results show a complex web of intersecting cultures such as a toxic macho culture, a weak leadership culture and a close relational culture that cooperate at different hierarchical levels and increase the risk for sexual harassment. The results also show how organizational factors such as workforce demography, unsocial working hours, staff turnover and understaffing are interacting with the organizational cultures in the creation of a hostile environment that increases the risk for sexual harassment.

**Conclusions:**

The results elucidate why traditional interventions such as training or bystander interventions are inefficient in the restaurant business. The results can be used to develop interventions that focus on macho-cultures in restaurants, the leadership culture and the specific relationship culture that develop due to the specific organizational structures in the restaurant business.

**Key messages:**

• There is a need to focus on organizational factors in order to work against sexual harassment in the restaurant business and is a prerequisite for developing efficient interventions.

• Sexual harassment affects many people in working life and is a serious public health problem. Also, sexual harassment at work maintains gender-based inequalities that exist at a structural level.

